# Wild pigs impact reproductive season movements and space use of wild turkeys

**DOI:** 10.1186/s40462-025-00578-x

**Published:** 2025-08-25

**Authors:** Travis E. Stoakley, Stephen J. Zenas, Vienna R. Brown, Mark. D. Smith, William D. Gulsby, Bret A. Collier, Stephen S. Ditchkoff

**Affiliations:** 1 College of Forestry, Wildlife and Environment, Auburn University, 602 Duncan Dr, Auburn, AL 36849 USA; 2 Warnell School of Forestry and Natural Resources, University of Georgia, 180 E Green St, Athens, GA 30602 USA; 3 Department of Agriculture, Animal and Plant Health Inspection Service, Wildlife Services, National Feral Swine Damage Management Program, National Wildlife Research Center, 4101 LaPorte Ave, Fort Collins, CO 80521 USA; 4 School of Renewable Natural Resources, Louisiana State University, Baton Rouge, LA 70803 USA

**Keywords:** Wild turkey, Wild pig, Resource selection function, Perceived risk, Interspecific interaction, Spatial interaction, Avoidance, Disturbance

## Abstract

**Background:**

Impacts of invasive species on the movements and space use of native fauna have potential implications during the reproductive period. Over the last three decades, native wild turkeys (*Meleagris gallopavo*) have experienced a notable decline in productivity while invasive wild pigs (*Sus scrofa*) have expanded populations throughout the southeastern United States. Camera studies have shown that the presence of wild pigs can negatively impact detection of wild turkeys in areas of overlapping use. We explored whether wild turkeys avoided areas with greater wild pig densities during spring breeding season for wild turkeys.

**Methods:**

We deployed 22 GPS units on wild turkeys prior to the spring reproductive season and conducted a 1-km^2^ gridded camera survey in early summer to estimate densities of wild pigs across our 9,000-ha study area in east-central Alabama. We addressed reproductive season movement ecology of wild turkeys in relation to relative densities of wild pigs in terms of (1) step length, (2) daytime space use, (3) roost site selection, and (4) nest site selection. We hypothesized that wild turkeys would exhibit longer step lengths and avoid daytime use, nighttime roost selection, and nest placement in areas with greater densities of wild pigs.

**Results:**

We found that greater densities of wild pigs negatively impacted movement metrics of wild turkeys. Specifically, greater densities of wild pigs were associated with longer step lengths and lower probabilities of daytime use, roost site selection, and nest site selection in wild turkeys.

**Conclusions:**

Rate of movement and probability of use are associated with preference for the ecological attributes of an area. Our results suggested that wild turkeys avoided or were excluded from areas with greater densities of wild pigs due to perceived disturbance risk or wild pigs making areas less usable. Our results have implications for interspecific spatial interactions as well as management activities to reduce the impacts of invasive wild pigs on native species.

## Background

Wild turkeys ( *Meleagris gallopavo* ) are an important cultural and ecological game species of North America. With hunting of wild turkeys generating an annual estimated $7 billion in economic activity in the United States (U.S.) [8], maintaining huntable populations remains a critical objective of wildlife management. Therefore, the documented decline in productivity of wild turkeys over the past several decades in the southeastern U.S. has been met with concern [19, 30]. Myriad factors have been suggested to contribute to diminished recruitment including decreased availability of suitable nesting and brooding habitat, overharvest through hunting activities, increases in predator populations, and interactions with introduced species [11, 14, 27, 80]. An introduced species of particular interest is the wild pig ( *Sus scrofa* ) which has seen pronounced increases in range and density across the southeastern U.S. over the past three decades [55]. Wild pigs have been documented to impact native fauna through nest depredation, disease transmission, competition for resources, degradation of habitat, direct predation, and potentially exclusionary behaviors due to perceived disturbance risk [26, 28, 40, 46, 55, 56, 64]. Due to the relative evolutionary novelty of wild pigs in the shared landscape with wild turkeys in our study area [55], we define perceived disturbance risk as a novel interspecific competitor that can be perceived by a competing species as a threat. In this case, the potential aggressive and exclusionary behaviors of wild pigs may be perceived as a threat by wild turkeys, leading to reduced use by wild turkeys of areas occupied by wild pigs. Perceived disturbance risk is of particular interest in the context of interspecific spatial interactions during reproductive periods due to potential negative downstream impacts on recruitment.

Native species can exhibit changes in fine-scale space use due to perceived risk from invasive competitors that subsequently impact population-level distribution patterns [66, 77]. Avoidance of encounters with invasive competitors such as wild pigs can result in shifts in patterns of resource selection [26, 28, 75]. Evidence suggests that wild pigs can impact species richness, resource availability, and spatiotemporal resource use by native fauna [37, 64]. Changes in rates and patterns of movement in relation to resource selection can also have fitness implications [6, 16, 38, 86]. For example, Byrne et al. (2022) found some preliminary evidence to suggest that female wild turkeys may nest in following years closer to nest sites that were successful [12]. Moreover, while Conley et al. (2016) did not find evidence to suggest habitat sampling by female wild turkeys during the pre-nesting period, over > 80% of all nest sites selected in the study were within the pre-nesting period range [21]. Additionally, multiple lines of evidence suggest that wild turkeys exhibit plasticity in broad-scale resource selection throughout the year [18], with hardwood and mixed forest cover important during fall and winter due to hard mast availability [36, 63, 65] and open cover important during spring for courting, nest site selection, and invertebrate availability [5, 13, 47]. Therefore, the potential of wild pigs to be perceived as risky by wild turkeys has potential to influence resource selection by making areas of importance to wild turkey ecology less usable.

Perceived risk may play a role in resource selection in the form of spatial exclusion, which was documented among Lord Howe Island woodhen (*Gallirallus sylvestris*) in Australia that shifted population-level spatiotemporal resource selection due to perceived risk from wild pigs [61]. Recent camera survey research has suggested that wild pigs potentially have negative spatiotemporal interactions with wild turkeys, seen in reduced detection rates of wild turkeys in areas with greater population indices of wild pigs [48, 56, 76]. Variation in observations of wild turkeys with respect to wild pig population indices could be driven by (1) wild turkey recruitment decreasing due to lower nest success or brood survival, or (2) wild turkeys avoiding or being excluded from areas with greater densities of wild pigs.

We evaluated whether wild turkeys avoided areas based on variation in density of wild pigs during the reproductive period for wild turkeys in east-central Alabama (1 March to 1 June 2022). We used a camera survey to estimate wild pig densities in areas where wild pigs and wild turkeys co-existed. We used GPS-tagged wild turkeys and developed resource selection functions (RSF) for wild turkey movements. Our research examined the relationship between relative densities of wild pigs and movement metrics of wild turkeys (including step length, daytime space use, roost site selection, and nest site selection). We hypothesized that areas with greater densities of wild pigs would observe greater step lengths and lower probabilities of daytime use, roost site selection, and nest site selection. Greater step lengths are associated with faster rates of movement [84], which could have implications for avoidance or escape behavior [49]. Reduced daytime use, roost site selection, and nest site selection could also indicate lower preference for areas with greater densities of wild pigs [9, 78].

### Study area

**Fig. 1:**
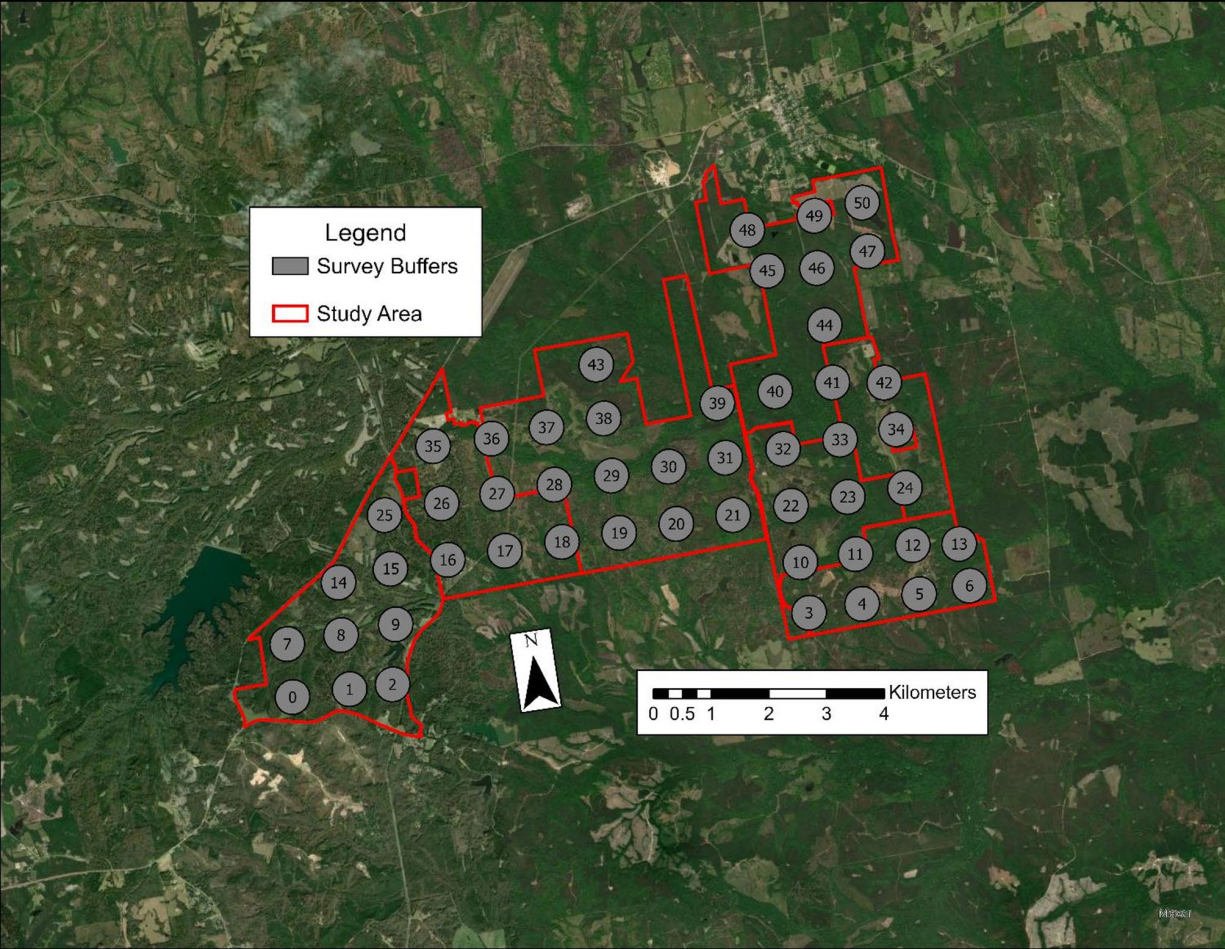
Study area map of seven adjoining properties in east-central Alabama with survey buffers denoted in grey circles and study area bounds outlined in red. The property in the southeastern corner (survey buffers 3–6 and 12–13) is surrounded by a high fence and is pig-free

Our study was conducted on 9,186 ha of contiguous privately-owned properties comprised of seven adjoining landowners. Located in the upper coastal plain in east-central Alabama, the study area was comprised of 5,562 ha of forest cover (60.5%), including 3,333 ha of pine (36.3%), 1,330 ha of hardwood (14.5%), and 899 ha of mixed pine-hardwood (9.8%). Open cover comprised 1,823 ha (19.9%) of the study area. The region had a subtropical climate with warm wet winters and hot humid summers (average annual temperature of 18 °C and approximately 133 cm of annual rainfall) [51]. Primary land management objectives on these properties were timber production and promotion of game species such as white-tailed deer (*Odocoileus virginianus*), northern bobwhite (*Colinus virginianus*), mourning dove (*Zenaida macroura*), and wild turkey. Forests were dominated by longleaf (*Pinus palustris*) and loblolly (*Pinus taeda*) pine, with intermixed hardwood stands of oaks (*Quercus* spp.), hickory (*Carya* spp.), maple (*Acer* spp.), and elm (*Ulmus* spp.) [30, 73]. Noteworthy, the 476 ha property in the southeastern corner of the study area (camera locations 3–6 and 10–12) was enclosed by an 2.5 m fence and had no wild pigs (Fig. 1).

### Methods

#### Deployment of GPS units on wild turkeys

We deployed 31 backpack-style GPS-VHF units (Lotek UK Ltd, Wareham, UK) on wild turkeys (13 males, 18 females) captured from five separate flocks with rocket nets over areas baited with cracked corn during January-March 2022 [4, 35]. Age was determined by presence of barring on ninth and tenth primary feathers and sex was determined by breast feather coloration [65]. Individuals were each outfitted with an aluminum rivet leg band (National Band and Tag Co., Newport, Kentucky, U.S.), and all capture and handling procedures were approved by Auburn University IACUC (PRN: 2021–3994). Each GPS-VHF unit was programmed to collect locations every two hours between 0600 and 2000 daily, with an additional point taken at 0000 for roost location. Units were also programmed to emit a mortality signal after 18 h of no detected movement. There were nine individuals (three males and six females) that died prior to data collection, two within the 14-day window of capture myopathy [10]. The remaining 22 wild turkeys (10 males and 12 females) were monitored weekly with Yagi antennas throughout the 1 March to 1 June 2022 study period.

#### Executing camera survey

We conducted a camera survey in May 2022 following methods described by Lewis et al. (2022) and McDonough et al. (2024) to estimate densities of wild pigs across the study area. A 1-km^2^ grid was applied over the study area in ArcGIS Pro™ (Esri, Redlands, California, U.S.) to determine locations for camera deployment. Grid cells that were < 25% within the bounds of the study area were excluded, creating 51 unique 1-km^2^ cells. A camera was placed within a 300-meter radius buffer of the center of each grid cell (Fig. 1). We used a camera spacing of 1-km^2^ for detection of wild pigs based on McDonough et al. (2024), a spacing which was also less than the typical home range size of wild pig sounders in the region [33, 48].

Camera sites were initially baited with 11 kg of whole corn and rebaited every 3–4 days throughout the 14-day camera survey period (first week pre-baiting and second week camera deployment and baiting. Cameras (ReconyxTM PC800 Hyperfire Professional IR Cameras, Reconyx Inc., Holmen, Wisconsin, U.S.) were deployed seven days after the initial establishment of bait sites. Cameras were oriented north-south on trees, one meter from the ground, and five meters from bait, with any visual obstruction removed. The cameras were programmed to take three images each time movement was detected with a one-minute buffer period between triggers. Cameras were deployed for seven days [89].

#### Estimating densities of wild pigs

We manually tagged images of wild pigs from the camera survey in Program TimeLapse2 V2.2.3.9 (University of Calgary, Calgary, CA). We estimated the density of wild pigs in each grid cell to be the total number of unique individuals per camera. Individuals were identified by size, sex, pelage, unique physical characteristics, sounder association, and non-overlapping timing of visitation [31]. Because the typical home range size of wild pig sounders in the region was larger than our camera spacing [32, 48], individual wild pigs could use more than one bait site, and unique individuals could thus be counted at multiple cameras. Because we were interested in estimating wild pig use in each grid cells, and counts in each grid cell could have included some wild pigs also counted in other grid cells, counts at each camera therefore determined the total number of individual wild pigs using a respective grid cell. The camera survey took place outside peak reproduction season for wild pigs and without perturbation of wild pig populations [25], so we assumed a stable local population for the three-month study period (1 March to 1 June 2022). We note that our timeline is also shorter than the length of gestation (~ 115 days) in wild pigs, so we assumed no major influence of reproduction on wild pig abundance [18, 36].

### Statistical analysis

**Fig. 2:**
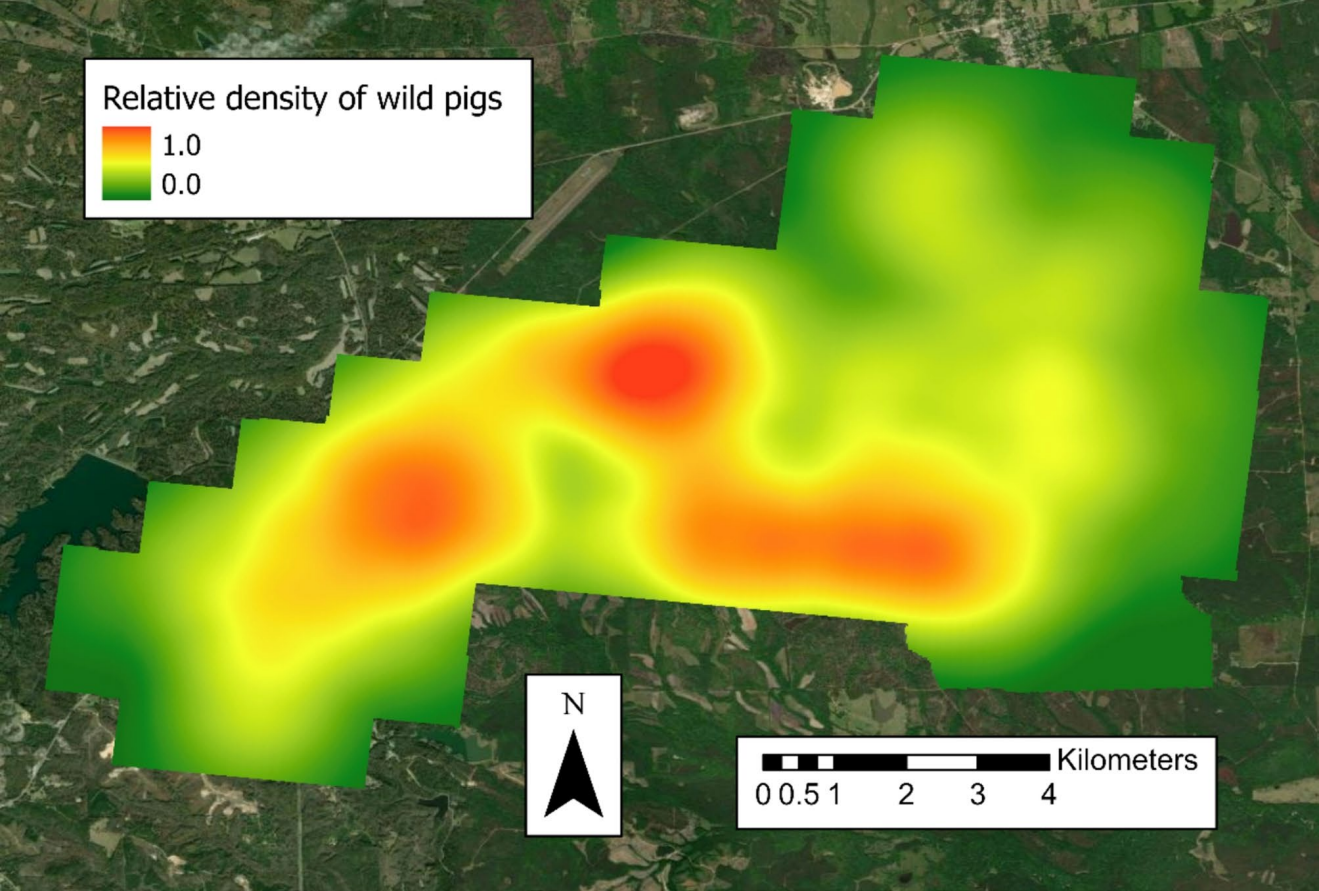
Relative density of wild pigs across buffered study area (0.0–1.0) in east-central Alabama in 30-m resolution for the May 2022 camera survey. Green denotes lower relative densities of wild pigs and red denotes greater relative densities of wild pigs

We standardized counts of wild pigs per cell across the 51 surveyed grid cells as relative densities per 1 km^2^. Relative density values were determined by ranking cell counts as percentiles (lowest = 0.0 and greatest = 1.0). Raw densities of wild pigs from the camera survey ranged from 0 to 42 pigs/km^2^ and were standardized to 0.0–1.0 pigs/km^2^. Each percentile was assigned to a respective grid cell. Relative densities were assigned in ArcGIS Pro™ in 1 km^2^ raster cells via the kernel density estimate tool. A 1 km buffer was created around the outside of the survey bounds to form the total study area, with buffer cell values determined as the average of adjacent survey cells [22, 54, 67]. No buffer was applied to cells bordering the high-fenced property (southeastern corner) because (1) the wild pig values within the fence were all zero and (2) the fence should not influence adjacent areas outside the fence (Fig. 2).

The camera and buffer values were re-interpolated in 30-m raster cells to match the resolution of the National Land Cover Data 2021 (NLCD 2021; rasterized satellite imagery for land cover composition that year) to standardize raster cell size (Fig. 1) [23].

We extracted land cover data at 30-m resolution from NLCD 2021 delineated as categories of pine forest, hardwood forest, mixed forest, open cover and riparian area. These land cover types had previously been determined to be biologically relevant cover types for wild turkey life history [15, 39]. Pine cover was defined as evergreen (pine) trees > 5 m tall that occupy > 20% of total vegetation cover, with > 75% of total tree cover present belonging to species that retain leaves year-round. Hardwood cover was defined as deciduous (hardwood) trees > 5-m tall that occupy > 20% of total vegetation cover, with > 75% of tree cover present belonging to species that lose leaves with seasonal change. Mixed cover was defined as having > 20% vegetation cover of trees > 5 m tall while neither evergreen nor deciduous species consist of > 75% of total tree cover. Forest cover types of pine, hardwood, and mixed forest were each coded as present (1) or absent (0). Open cover types in NLCD 2021 of cropland, grassland, shrub, and road were combined as Open and coded as present (1) or absent (0). Riparian cover type was classified as present (1) or absent (0) within a 100 m buffer of water or wetland area. We used these land cover variables as interactions with wild pig density to determine the magnitude of effect wild pigs had on probability of use by wild turkeys in each cover type.

Movement data for wild turkeys was cleaned to remove erroneous fix locations with dilution of precision (DOP) > 7 and points outside the study area [34]. Movement data were then grouped into the following categories: ALL, MALES, BREEDING FEMALES, and NONBREEDING FEMALES. Females were categorized as BREEDING FEMALES from the start of the reproductive season (March 1) until the individual termination of nesting or brooding [83, 87]. Females were categorized as NONBREEDING FEMALES from the first full day of inactivity from nesting or brooding to the end of the survey period (June 1). Step length and utilization distribution metrics for resource selection were determined via dynamic Brownian bridge movement models in package move in Program R [45, 72]. Step length was calculated as the Pythagorean distance between two consecutive points. Resource selection was evaluated via second-order selection of all point by grouping within the bounds of the study area (e.g., all daytime space use points for BREEDING FEMALES grouping) [42, 53]. We used a generalized linear model with a Gaussian distribution to examine the step length (log-transformed dependent variable) in relation to relative densities of wild pigs (predictor variable) via the stats package in Program R. Step length calculations were extracted for step-to locations. The predictor variable of relative wild pig density was extracted to points by grouping (i.e., ALL, MALES, BREEDING FEMALES, and NONBREEDING FEMALES). Land cover variables were not included in this portion of the analysis because line segments could cross multiple cover types in each step. The model formula was conceptualized as follows: Log(Step Length) = β_0_ + β_1_*(Relative Wild Pig Density). We then exponentiated the log-transformed effect sizes from the model to obtain values on the linear scale.

We examined impacts of wild pigs and interactions with land cover type on daytime use by using an RSF for known and random daytime use points. We created 100,000 random points within the MCP of all daytime use points within the study area for each grouping (i.e., ALL, MALES, BREEDING FEMALES, and NONBREEDING FEMALES) via the create random points function in ArcGIS Pro with a minimum spacing of 10 m. We employed the model selection methodology of Bakner et al. (2024) for inclusion of land cover types with known importance in wild turkey ecology. Predictor variables of relative wild pig density and land cover were extracted to both used and available daytime locations. We used a generalized linear model with a binomial distribution (logit-link) via the stats package in Program R for the predicted probability of daytime use (dependent variable) for interactions between relative wild pig density and land cover types of hardwood forest, mixed forest, open, pine forest, and riparian cover (predictor variables). We assigned used points a value of 1 and random points a value of 0 to create a used-available matrix within the MCP of all daytime use points within the study area for each grouping of wild turkeys. The model formula was conceptualized as follows: Used/Available = β_0_ + β_1_*(Relative Wild Pig Density*Hardwood Forest) + β_2_*(Relative Wild Pig Density*Mixed Forest) + β_3_*(Relative Wild Pig Density*Open) + β_4_*(Relative Wild Pig Density*Pine Forest) + β_5_*(Relative Wild Pig Density*Riparian). We additionally checked whether individual variation in the number of points collected per individual biased model prediction in the daytime use analysis by using generalized linear mixed-effect models in the glmmTMB package in Program R. However, we did not find any biologically significant differences in our predictions across models, so we maintained reporting of GLM outputs in the results section.

We examined impacts of wild pigs and interactions with land cover type on roost site selection via an RSF for known and random nighttime roost sites. Roost site locations were selected as midnight locations [3, 20]. We created 100,000 random points within the MCP of all roosting sites within the study area for each grouping (i.e., ALL, MALES, BREEDING FEMALES, and NONBREEDING FEMALES) via the create random points function in ArcGIS Pro with a minimum spacing of 10 m. Predictor variables of relative wild pig density and land cover variables were extracted to both used and available roost locations. Again, we employed the model selection methodology of Bakner et al. (2024) for inclusion of land cover types with known importance in wild turkey ecology. We used a generalized linear model with a binomial distribution (logit-link) for the predicted probability of roost site selection (dependent variable) for interactions between relative wild pig density and land cover types of hardwood forest, mixed forest, pine forest, and riparian cover (predictor variables). We assigned used points a value of 1 and random points a value of 0 to create a used-available matrix within the MCP of all roost sites within the study area for each grouping of wild turkeys. The model formula was conceptualized as follows: Used/Available = β_0_ + β_1_*(Relative Wild Pig Density*Hardwood Forest) + β_2_*(Relative Wild Pig Density*Mixed Forest) + β_3_*(Relative Wild Pig Density*Pine Forest) + β_4_*(Relative Wild Pig Density*Riparian). Open cover type was included for only BREEDING FEMALES because female wild turkeys roost on the ground during nesting and early brooding activities [13, 47, 74]. Therefore, the model formula was conceptualized as follows: Used/Available = β_0_ + β_1_*(Relative Wild Pig Density*Hardwood Forest) + β_2_*(Relative Wild Pig Density*Mixed Forest) + β_3_*(Relative Wild Pig Density*Open) + β_4_*(Relative Wild Pig Density*Pine Forest) + β_5_*(Relative Wild Pig Density*Riparian). We additionally checked whether individual variation in the number of points collected per individual biased model prediction in the roost site selection analysis by using generalized linear mixed-effect models in the glmmTMB package in Program R. However, we did not find any biologically significant differences in our predictions across models, so we maintained reporting of GLM outputs in the results section.

During the reproductive season, females were monitored twice per week to determine nesting activity. If actively nesting, characterized by the grouping of points in a singular location (Bakner et al. 2024), then females were checked daily with VHF to monitor nest fate. Nests were checked in-person within 24 h of nest termination or after 28 days post initiation of incubation. There were 16 nests within the study area that were verified in-person. Two nests successfully hatched poults which were verified in-person as having the presence of eggshell pipping. We created 100,000 random points within the MCP of all nesting sites of BREEDING FEMALES within the study area via the create random points function in ArcGIS Pro with a minimum spacing of 10 m. Predictor variables of relative wild pig density and land cover were extracted to both used and available nest locations. We used a generalized linear model with a binomial distribution (logit-link) for the predicted probability of nest site selection (dependent variable) in relation to relative wild pig density (predictor variable). Land cover interactions were not included due to low sample size of nest sites. The model formula was conceptualized as follows: Used/Available = β_0_ + β_1_*(Relative Wild Pig Density).

## Results

A total of 22 wild turkeys (10 males, 12 females) were monitored during the 1 March to 1 June 2022 study period. All 12 females nested and thus were classified as BREEDING FEMALES from the beginning of the study period (1 March 2022) until the cessation of individual nesting or brood rearing. One female was predated while nesting, so we classified 11 females as NONBREEDING FEMALES from the first day post individual nesting or brooding until the end of the study period (1 June 2022). For daytime use, we recorded 12,266 known points for ALL, 5,936 known points for BREEDING FEMALES, 2,282 known points for NONBREEDING FEMALES, and 4,048 known points for MALES. The average relative wild pig density for daytime use of the ALL grouping was 27.5% lower for used pointed (0.271) than random points (0.374). For roost site selection, we recorded 1,778 known points for ALL, 857 known points for BREEDING FEMALES, 329 known points for NONBREEDING FEMALES, and 592 known points for MALES. The average relative wild pig density for roost site selection of the ALL grouping was 34.2% lower for used pointed (0.253) than random points (0.384).

Relative density of wild pigs was positively associated with step length for ALL grouping, NONBREEDING FEMALES, and MALES (Table 1).

**Table 1:** Change in step length (β in meters) per 1% increase in relative density of wild pigs (*Sus scrofa*) by grouping of wild turkey (*Meleagris gallopavo*) during reproductive season (1 March to 1 June 2022) in east-central alabama. The ALL grouping consisted of 22 wild turkeys, BREEDING FEMALES had 12 individuals, NONBREEDING FEMALES had 11 individuals, and MALES had 10 individuals

Grouping	β	*P*	95% Confidence Interval
ALL	1.29	< 0.001	1.20–1.39
BREEDING FEMALES	1.00	0.978	0.81–1.24
NONBREEDING FEMALES	2.55	< 0.001	2.24–2.89
MALES	1.17	0.009	1.04–1.32

The average step length was 253.5 m for the ALL grouping, 231.8 for BREEDING FEMALES, 216.6 for NONBREEDING FEMALES, and 288.5 m for MALES. For every 10% increase in relative wild pig density, we observed a 12.9 m increase in step length for the ALL grouping (*P* < 0.001), a 25.5 m increase in step length for NONBREEDING FEMALES (*P* < 0.001), and a 11.7 m increase in step length for MALES (*P* = 0.009).

**Fig. 3:**
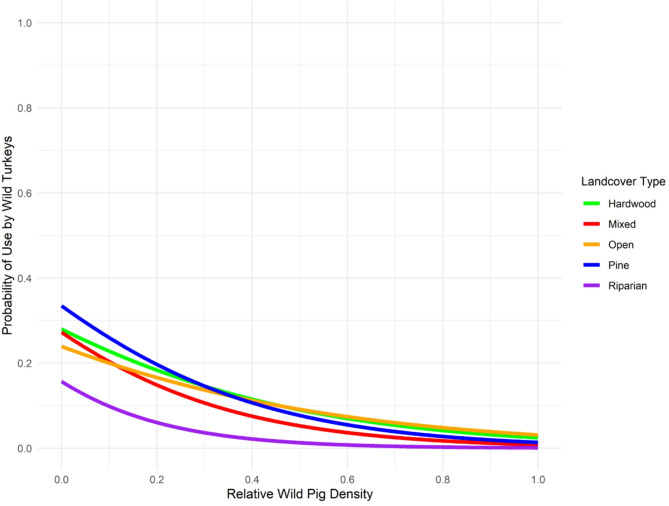
Predicted probability of daytime space use (0.0–1.0) by land cover interaction for the ALL grouping of wild turkeys (*Meleagris gallopavo*) by relative density quantile (0.0–1.0) of wild pigs (*Sus scrofa*) during reproductive season (1 March to 1 June 2022) in east-central Alabama. Hardwood forest cover was denoted in green, mixed forest cover was denoted in red, pine forest cover was denoted in blue, and riparian cover was denoted in purple

We observed a negative relationship between wild pig density and probability of daytime use by wild turkeys for the ALL grouping (β = -2.73, 95%: -2.97 – -2.49, *P* < 0.001), BREEDING FEMALES (β = -2.55, 95% CI: -2.86 – -2.24, *P* < 0.001), NONBREEDING FEMALES (β = -2.13, 95% CI: -2.80 – -1.47, *P* < 0.001), and MALES (β = -5.10, 95% CI: -5.57 – -4.63, *P* < 0.001). When examining interactions between land cover covariates and wild pig density, we also found a stepwise reduction in predicted probability of daytime use by grouping as wild pig density increased (Table 2; Fig. 3).

**Table 2:** Predicted probability of daytime space use for groupings of wild turkey (*Meleagris gallopavo*) for relative density quantile of wild pigs (*Sus scrofa*) by land cover interaction during reproductive season (1 March to 1 June 2022) in east-central alabama. The ALL grouping consisted of 22 wild turkeys, BREEDING FEMALES had 12 individuals, NONBREEDING FEMALES had 11 individuals, and MALES had 10 individuals

Grouping	Wild pig density quantile	Pig*Pine	Pig*Hardwood	Pig*Mixed	Pig*Riparian	Pig*Open
ALL	0.00	0.335	0.280	0.272	0.157	0.239
	0.25	0.170	0.164	0.126	0.047	0.151
	0.50	0.077	0.090	0.053	0.013	0.091
	0.75	0.033	0.048	0.021	0.003	0.054
	1.00	0.014	0.025	0.008	0.001	0.031
BREEDING FEMALES	0.00	0.149	0.171	0.134	0.099	0.198
	0.25	0.084	0.099	0.065	0.019	0.104
	0.50	0.046	0.055	0.031	0.003	0.052
	0.75	0.025	0.030	0.014	0.001	0.025
	1.00	0.013	0.016	0.007	< 0.001	0.012
NONBREEDING FEMALES	0.00	0.085	0.040	0.077	0.291	0.054
	0.25	0.035	0.024	0.031	0.039	0.033
	0.50	0.014	0.014	0.012	0.004	0.021
	0.75	0.006	0.008	0.005	< 0.001	0.013
	1.00	0.002	0.005	0.002	< 0.001	0.008
MALES	0.00	0.265	0.176	0.234	0.030	0.128
	0.25	0.073	0.056	0.050	0.004	0.054
	0.50	0.017	0.016	0.009	< 0.001	0.021
	0.75	0.004	0.005	0.002	< 0.001	0.008
	1.00	0.001	0.001	< 0.001	< 0.001	0.003

From lowest to greatest relative wild pig density (0.0–1.0) for the ALL grouping of wild turkeys, the predicted probability of daytime use decreased by an overall 32.1% in pine cover, 25.5% in hardwood cover, 26.6% in mixed cover, 15.6% in open cover, and 20.8% in riparian cover. From lowest to greatest relative wild pig density (0.0–1.0) for BREEDING FEMALES, the predicted probability of daytime use decreased by an overall 13.6% in pine cover, 15.5% in hardwood cover, 12.7% in mixed cover, 9.8% in riparian cover, and 18.6% in open cover. From lowest to greatest relative wild pig density (0.0–1.0) for NONBREEDING FEMALES, the predicted probability of daytime use decreased by an overall 8.3% in pine cover, 3.5% in hardwood cover, 07.5% in mixed cover, 29.8% in open cover, and 4.6% in riparian cover. From lowest to greatest relative wild pig density (0.0–1.0) for MALES, the predicted probability of daytime use decreased by an overall 26.4% in pine cover, 17.5% in hardwood cover, 23.3% in mixed cover, 2.9% in open cover, and 12.5% in riparian cover.

**Fig. 4:**
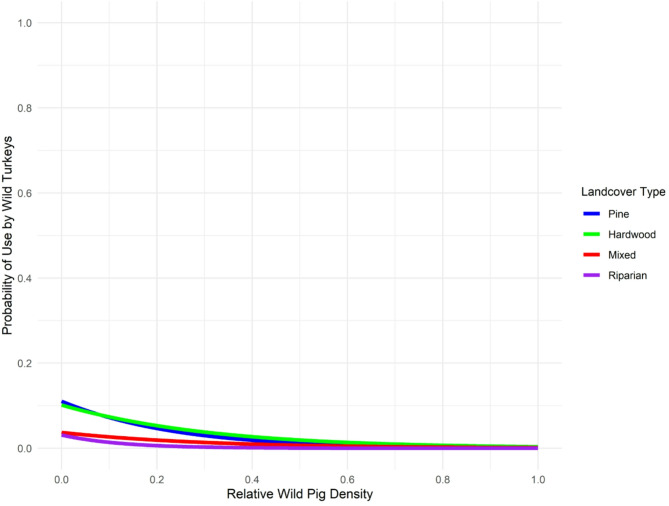
Predicted probability of roost site selection (0.0–1.0) by land cover interaction for the ALL grouping of wild turkeys (*Meleagris gallopavo*) by relative density quantile (0.0–1.0) of wild pigs (*Sus scrofa*) during reproductive season (1 March to 1 June 2022) in east-central Alabama. Hardwood forest cover was denoted in green, mixed forest cover was denoted in red, pine forest cover was denoted in blue, and riparian cover was denoted in purple

Probability of roost site selection was negatively related to wild pig density for the ALL grouping (β = -3.50, 95% CI: -4.05 – -2.97, *P* < 0.001), BREEDING FEMALES (β = -3.74, 95% CI: -4.46 – -3.04, *P* < 0.001), and MALES (β = -6.00, 95% CI: -7.16 – -4.86, *P* < 0.001). When examining interactions between land cover covariates and wild pig density, we also found a stepwise reduction in predicted probability of roost site selection by grouping as wild pig density increased (Table 3; Fig. 4).

**Table 3:** Predicted probability of roost site selection for groupings of wild turkey (*Meleagris gallopavo*) for relative density quantile of wild pigs (*Sus scrofa*) by land cover interaction during reproductive season (1 March to 1 June 2022) in east-central alabama. The ALL grouping consisted of 22 wild turkeys, BREEDING FEMALES had 12 individuals, NONBREEDING FEMALES had 11 individuals, and MALES had 10 individuals

Grouping	Wild pig density quantile	Pig*Pine	Pig*Hardwood	Pig*Mixed	Pig*Riparian	Pig*Open
ALL	0.00	0.110	0.102	0.037	0.031	NA
	0.25	0.037	0.045	0.016	0.004	NA
	0.50	0.012	0.019	0.007	0.001	NA
	0.75	0.004	0.008	0.003	< 0.001	NA
	1.00	0.001	0.003	0.001	< 0.001	NA
BREEDING FEMALES	0.00	0.039	0.070	0.019	0.026	0.020
	0.25	0.016	0.028	0.009	0.001	0.008
	0.50	0.007	0.011	0.004	< 0.001	0.003
	0.75	0.003	0.004	0.002	< 0.001	0.001
	1.00	0.001	0.002	0.001	< 0.001	0.001
NONBREEDING FEMALES	0.00	0.023	0.005	0.009	0.173	NA
	0.25	0.009	0.004	0.004	0.004	NA
	0.50	0.003	0.003	0.002	< 0.001	NA
	0.75	0.001	0.002	0.001	< 0.001	NA
	1.00	< 0.001	0.002	< 0.001	< 0.001	NA
MALES	0.00	0.085	0.040	0.077	0.291	NA
	0.25	0.035	0.024	0.031	0.039	NA
	0.50	0.014	0.014	0.012	0.004	NA
	0.75	0.006	0.008	0.005	< 0.001	NA
	1.00	0.002	0.005	0.002	< 0.001	NA

From lowest to greatest relative wild pig density (0.0–1.0) for the ALL grouping of wild turkeys, the predicted probability of roost site selection decreased by an overall 10.9% in pine cover, 9.9% in hardwood cover, 3.6% in mixed cover, and 3.0% in riparian cover. From lowest to greatest relative wild pig density (0.0–1.0) for BREEDING FEMALES, the predicted probability of roost site selection decreased by an overall 2.8% in pine cover, 6.8% in hardwood cover, 1.8% in mixed cover, 2.5% in riparian cover, and 1.9% in open cover. From lowest to greatest relative wild pig density (0.0–1.0) for NONBREEDING FEMALES, the predicted probability of roost site selection decreased by an overall 2.2% in pine cover, 0.3% in hardwood cover, 0.8% in mixed cover, and 17.2% in riparian cover. From lowest to greatest relative wild pig density (0.0–1.0) for MALES, the predicted probability of roost site selection decreased by an overall 8.3% in pine cover, 3.5% in hardwood cover, 7.5% in mixed cover, and 29.0% in riparian cover.

**Fig. 5:**
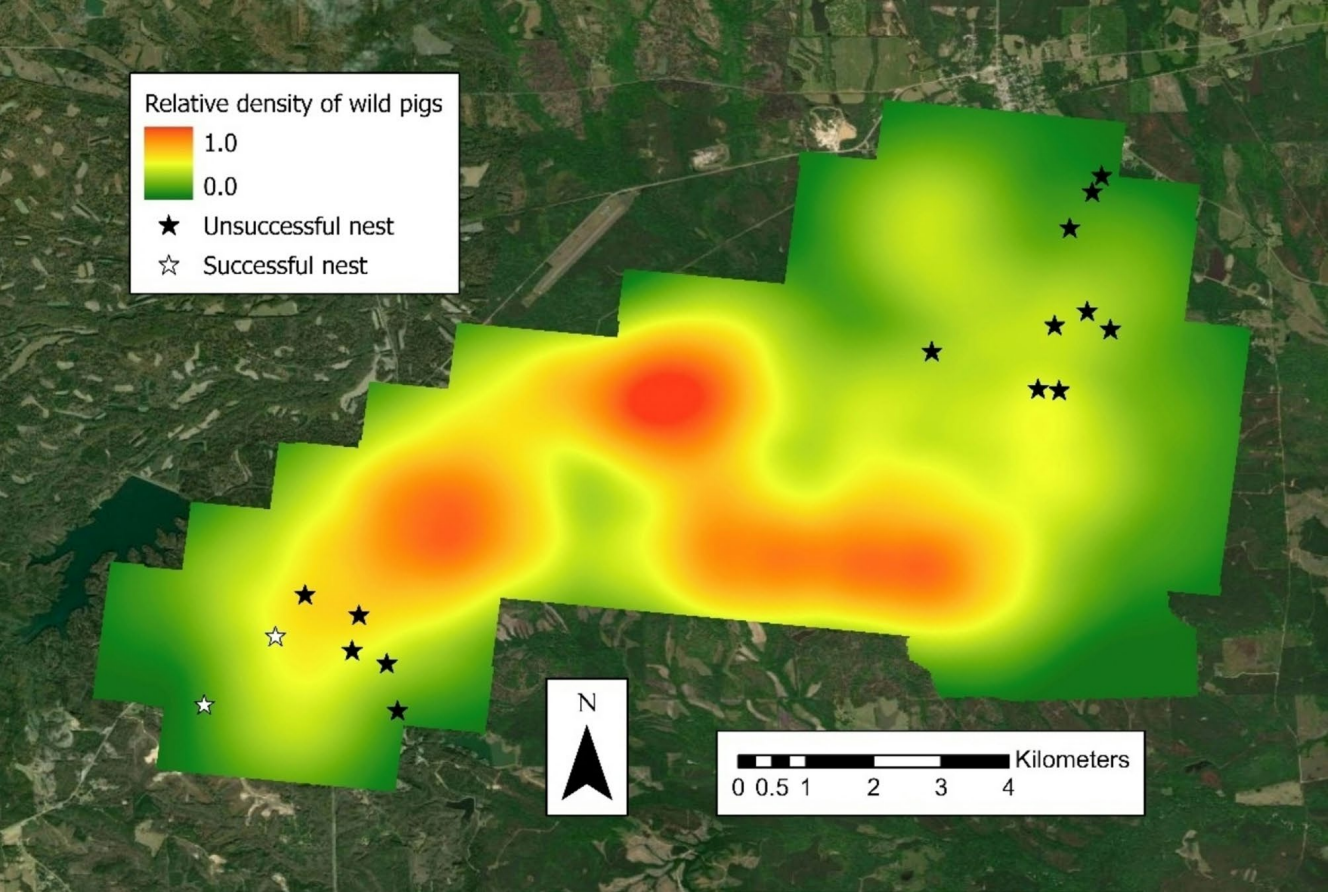
Nest locations of 16 wild turkeys (*Meleagris gallopavo*) in east-central Alabama during the reproductive season (1 March to 1 June 2022) in relation to relative density of wild pigs (*Sus scrofa*) from the May 2022 camera survey at 30-m resolution. Lowest relative densities of wild pigs (0.0) are denoted in green and greatest relative densities of wild pigs (1.0) are denoted in red. Unsuccessful nests are denoted as black stars and successful nests are denoted as white stars

A total of 16 nests were initiated by the 12 females in the BREEDING FEMALES grouping, as renesting occurred (Fig. 5).

The average relative density of wild pigs was 0.31 for known sites and 0.41 for random nest sites, with the two successful nests respectfully at 0.14 and 0.44. Additionally, nest site selection by BREEDING FEMALES was negatively related to relative wild pig density (β = -6.22, 95% CI: -9.61 – -2.85, *P* = 0.007).

## Discussion

We found that wild pigs had a range of predicted negative impacts on wild turkeys, suggesting a potential for wild pig density to influence movements and space use of wild turkeys. Namely, we observed evidence of variation in metrics of step length, daytime space use, roost site selection, and nest site selection related to wild pig density. Our results suggest that selection by wild turkeys for areas with lower densities of wild pigs indicated that (1) wild pigs were perceived as disturbance risk or (2) areas with greater densities of wild pigs were perceived as less favorable. Furthermore, while wild pigs may not serve as predators of adult wild turkeys, we posit that the drivers of differences in space use by wild turkeys were (1) inherent avoidance of areas associated with wild pigs or (2) exclusion from areas by wild pigs due to perceived risk of disturbance.

Whereas temporal avoidance or exclusion by wild pigs has been proposed for wild turkeys in previous camera survey studies [58, 76, 85], a driver of variability or an explanation of spatial impacts has never been explored. The relationship between use of an area and selection for ecological attributes of that area is established in ecological theory [59, 68]. There is also precedent that areas avoided contain ecological attributes that are less favorable or invoke perceived risk [9, 78]. Inherent avoidance is supported in the context of wild pigs and wild turkeys by Walters and Osbourne (2021) in which rates of detection for wild turkeys decreased in areas of overlapping use with wild pigs. Prior research has also found that wild turkeys disproportionally used areas with reduced risk of predation [83, 91]. We also recognize the relative novelty of wild pigs on the landscape around our study area on an evolutionary timeline [55, 56], suggesting that wild turkeys may perceive wild pigs as a disturbance risk due to unfamiliarity. Again, while wild pigs may not pose a threat to adult wild turkeys in the form of predation, we believe the wild turkeys in our study perceived wild pigs as disturbance risk, leading to spatiotemporal avoidance or exclusion from areas of overlapping use.

We found that step lengths of wild turkeys were greater in areas with greater densities of wild pigs for the ALL, NONBREEDING FEMALES, and MALES groupings. Step length acts as an indicator for rate of movement and increases in rates of movement can be associated with avoidance behaviors from perceived risks [49, 81, 90]. Pusenius et al. (2020) suggested that moose (*Alces alces*) adjusted rates of movement in relation to presence of grey wolves (*Canis lupus*) [70]. Similarly, Laundré et al. (2001) suggested that elk (*Cervus elaphus*) and bison (*Bison bison*) displayed behavioral responses to the presence of wolves that exceeded actual risk of predation. While not driven by risk of predation, we believe that wild turkeys exhibited disturbance-driven risk avoidance behaviors by increasing rates of movement in areas with greater densities of wild pigs. By moving quicker through areas of perceived risk, wild turkeys spent less time potentially exposed to disturbance threats associated with wild pigs. This potentially has downstream implications for condition in terms of energy use, feeding activity, and vulnerability to predation, as well as recruitment in terms of breeding activity and brood rearing. We also recognize the importance of land cover differences and movement corridors as drivers of movement rate, and we believe that data with greater temporal resolution (e.g. more frequent than two-hour locational fixes) could potentially account for land cover relationships with step length.

We found a series of negative effects of wild pig density on movement rates of wild turkeys; however, whether these effects have biological significance is unknown. Step length serves as a representation of how space use affects daytime biological operations, including foraging efficiency and awareness of predatory threats [81]. An aspect of optimal foraging theory focuses on how rate of movement influences foraging efficiency, and variations from a given movement rate with respect to the optimal would result in reduced individual fitness [71]. Elevated movement rates due to risk aversion are associated with reduced foraging efficiency [49, 90]. Increased movement rates of turkeys in our study could theoretically have resulted in reduced foraging efficiency due to perceived disturbance risk by wild pigs. Step length is measured as linear distance [81], and animals that are restricted to more linear movements may have enhanced risk of predation [29, 41, 90]. Adam and Stuart-Smith (2000) found that woodland caribou (*Rangifer tarandus*) had greater rates of predation when restricted to linear corridors. Similarly, Prokopenko et al. (2016) found that increases in linear movements by red deer (*Cervus elaphus*) resulted in greater rates of predation [69]. Therefore, the influence of wild pigs to restrict movement patterns of wild turkeys via perceived disturbance risk may expose wild turkeys to greater risk of predation to predators such as bobcats and coyotes [60]. Increased linearity of movement is also associated with faster rates of movement [24, 52]. Thus, wild pigs may have influenced both the linearity and speed of movements of wild turkeys, resulting in longer step lengths through areas of greater wild pig density.

We observed wild turkeys exhibiting less daytime space use in areas with greater densities of wild pigs. A negative relationship between temporal use by wild turkeys in relation to wild pig density has been supported by several camera survey studies [40, 56, 76, 85]. While these studies reported that wild pigs decreased detection or temporally displaced wild turkeys, our results indicate that the heterogeneity of space use by wild turkeys was influenced by the heterogeneity of wild pig density across the study area. We believe that wild turkeys either deliberately avoided areas associated with wild pigs or were actively excluded from areas by wild pigs. While we observed numerous negative interactions between wild pig density and land cover variables for probability of daytime use across groupings, we also found that wild turkeys in the ALL, BREEDING FEMALES, AND MALES groupings did not select against open cover in association with wild pig density. We believe that the value of open cover during the reproductive period may exceed the perceived disturbance risk incurred with wild pigs. Open areas are important for courting displays by male wild turkeys [44, 79], as well as valuable foraging areas during the reproductive period [62, 79]. Therefore, when individuals became reproductively inactive (e.g. NONBREEDING FEMALES), the perceived disturbance risk associated with wild pigs manifested in a negative association with probability of daytime use in open cover. With open cover limited in availability in the study area, there were also no alternatives to openings used by wild pigs.

We also found a decrease in probability of roost site selection across each grouping of wild turkeys in relation to density of wild pigs. Suitable roost sites are important in wild turkey ecology to mitigate environmental exposure or risk of predation, which is greatest during crepuscular periods when roost sites are selected [1, 3, 7]. Wild pigs are highly active during crepuscular and nighttime periods [19], so avoidance of areas where wild pigs are present during these periods could mitigate perceived disturbance risk [28, 78]. Furthermore, avoidance of areas with greater densities of wild pigs during the daytime would likely lead to avoidance when selecting a place to roost as well. Similarly, roosting in areas with lower densities of wild pigs would likely lead to daytime use of areas with lower densities of wild pigs. Previous studies have found that condition and survival of wild turkeys has been linked to disproportionate roost selection near supplemental food sources or areas that reduced risk of environmental exposure or predation [43, 57]. Adey et al. (2023) suggested that avoidance of predation risk was the primary factor driving roost site selection for wild turkeys in Canada. Similarly, we believe that the wild turkeys in our study selected against roost sites located in areas with greater densities of wild pigs to reduce risk of disturbance by wild pigs. We posit that this was due to wild turkeys perceiving wild pigs as threats during vulnerable crepuscular periods in which roost sites were selected.

Wild turkey females disproportionally nested in areas with lower densities of wild pigs as well. We believe that these results indicated some evidence that nesting females may perceive wild pigs as a threat to reproductive success. The most vulnerable time during the life history of a mature female wild turkey is during the four-week nest incubation and first two weeks of brood rearing in which females roost on the ground [13, 50, 59]. Selecting a nest site that minimizes perceived disturbance risk would best serve to promote reproductive success. Therefore, nesting in areas with lesser densities of wild pigs would serve to minimize risk of disturbance while nesting. Our study observed an above average rate of nest failure (87.5%, average = 59–76%) [22, 91] and brood failure (100%, average = 64–76%) [2, 91] for wild turkeys in the southeastern U.S. This could indicate that the female wild turkeys in our study area may be selecting less suitable areas to nest (1) to minimize risk of encounters with wild pigs, or (2) due to exclusion from more suitable nesting grounds by wild pigs. Nesting in less suitable areas can potentially expose nesting females to greater rates of nest failure due to predation or environmental exposure [5, 11]. Ulrey et al. (2022) examined predator populations in relation to wild turkey reproduction and found greater abundance indices of wild pigs in areas with unsuccessful wild turkey nests [82]. Our results are particularly important for wild turkeys in the southeastern U.S when we consider declining rates of productivity and recruitment, as well as diminishing quality of nesting and brooding habitat [17].

While wild pigs may not pose a meaningful predatory threat to mature wild turkeys, our findings suggest that wild pigs may influence the movement ecology or resource selection of wild turkeys during breeding season. We believe that wild turkeys perceived encounters with wild pigs as disturbance risk and selected for areas with lower densities of wild pigs to reduce these encounters. With this, we postulate that areas with greater densities of wild pigs were perceived as less favorable and therefore became less usable to wild turkeys. We also propose that a possible driver of differences in space use observed by wild turkeys could have been avoidance of areas associated with wild pigs or exclusion from areas by wild pigs.

## Conclusion

Our study provides an approach to explaining an aspect of the spatiotemporal relationship between an ecologically impactful invasive large mammal and an economically important native ground-nesting bird. Through the application of an RSF to camera trapping of wild pigs and GPS-VHF movement data of wild turkeys, we described the impact of perceived disturbance risk of wild pigs by wild turkeys. Invasive species pose the second greatest threat to biodiversity across the globe [88], with wild pigs acting as a major threat to declining and imperiled species [54]. The impacts of wild pigs on the movements of native species likely does not end with the spring reproductive season of wild turkeys, so future work should continue to examine impacts on movement, reproduction, and survival of declining or threatened species that share overlapping ranges with wild pigs.

## Data Availability

The datasets used in the analysis for this study are available from the corresponding author on reasonable request, as well as from the Harvard Dataverse repository.

## Abbreviations

DOP: dilution of precision
GPS: Global Positioning System
MCP: minimum convex polygon
NLCD: National Land Cover Data
RSF: resource selection function
U.S.: United States
VHF: very high frequency
